# Comprehension and storage of sequentially presented radio news items by healthy elderly

**DOI:** 10.1590/S1980-57642009DN30200009

**Published:** 2009

**Authors:** Milena Vaz Bonini, Letícia Lessa Mansur

**Affiliations:** 1 Monografia de conclusão do Curso de Fonoaudiologia da FMUSP; 2 Professora Associada do Curso de Fonoaudiologia da FMUSP, São Paulo SP, Brazil.

**Keywords:** language, elderly, cognition

## Abstract

In the normal aging processes, complaints of memory loss and auditory comprehension are not uncommon, principally in complex and cognitively demanding situations.

**Objective:**

The objective of this study was to investigate skills of discourse comprehension and retention in a natural situation, by healthy aged in relation to variables such as age, schooling and cognitive screening measurement.

**Methods:**

Thirty healthy elderly participated in the study (mean age=73.56 yrs; SD=6.26 and mean schooling=8.6 yrs; SD=4.41). Twelve news items were recorded and presented in three sequences of four news-groups. Participants were instructed to listen to the four news items, and upon completion were questioned about one of them.

**Results:**

We found no age or schooling effect on the performance of the subjects. The participants achieved almost full scores on all answers (ceiling effect).

**Discussion:**

The heterogeneity of elderly and cognitive compensation in natural situations could explain these results of elderly behavior.

## Abstract (pt)

No processo de envelhecimento, queixas sobre declínio de memória e dificuldades de compreensão auditiva não são raras, principalmente em situações complexas e com maior demanda cognitiva.

**Objetivos:**

O objetivo do estudo foi investigar habilidades de compreensão e retenção de discurso por idosos, em situações naturais, considerando as variáveis: idade, escolaridade e medida cognitiva.

**Métodos:**

30 idosos participaram do estudo (média de idade=73,56; desvio padrão=6,26 e média de escolaridade=8,6; desvio padrão=4.41). Doze noticias foram gravadas e apresentadas em três seqüências de quatro grupos cada. Os participantes foram instruídos a ouvir as seqüências e ao final foram questionados sobre uma das notícias.

**Resultados:**

Não foram encontrados efeitos de idade ou escolaridade na perfórmance dos participantes. Notou-se efeito teto em todos os escores.

**Discussão:**

A heterogeneidade do envelhecimento e compensações cognitivas em situações naturais poderiam explicar o comportamento dos idosos da amostra.

Numerous studies have associated aging to cognitive decline, where this deterioration leads to difficulties in processing, especially of complex information such as texts.^1-3^

In the normal aging process, memory decline is a frequent complaint. Difficulties reported include retention of verbal content, such as remembering names of family members, and information pertaining to texts read or content heard. Recent research in older listeners have revealed difficulties when engaged in complex tasks involving the auditory processing of naturalistic signals in realistic social and physical environments. Tasks affected include discourse activity in conversation, or following a story in a book or on television, in which recognition, comprehension and storage of the material is impacted.^4^

Regarding the relationship between memory and language, most of the studies have evaluated the memorization of word lists.^5,6^ The retention and comprehension of discourse involves diverse mechanisms, whereby macro-structures determine a semantic axle of principal information to which others, secondary or accessory, are related either directly or indirectly.^7-9^ On the other hand, retention and comprehension of information from a discourse requires the participation of working memory in multiple degrees of processing such as lexical, syntactic or semantic, in order to obtain the sense of the discourse. The complexity of the task in natural situations (ecological) involves additional challenges, because frequently events occurs in situations of time pressure, as is the case with the news on the radio, in which the broadcaster transmits the message in a sequence of chained facts, read out, except in special conditions, without redundancies.

The study of Yasuda, Nakamura and Beckman^10^ investigated skills of discourse comprehension and retention in the older and aphasic population. The task used was to hear one news report and three sequences composed of four news items each and to later answer questions related to the news heard. It was shown that discourse retention seems to obey hierarchic importance with the main ideas occupying higher levels and details at the lowest levels. It was also noted that when comprehension and storing of information are simultaneously solicited in a task, aging presented preservation of comprehension yet loss of storage.

Among the many studies related to discourse comprehension and storing in aging, the work by Ryan is noteworthy.^11^ This author defends the position that the performance of the aged is related to their level of schooling, the effort demanded by the task, material organization, high demand on working memory and free recall of discourse.

Based on the points above, the objective of this study was to investigate skills of discourse comprehension and retention in a natural situation, by healthy aged in relation to variables such as gender, age and schooling.

## Methods

### Participants

Thirty healthy elderly participated in the study. Subjects signed a Term of Consent and were selected according to the following inclusion criteria: score less than or equal to 10 on the Geriatric Depression Scale (GDS),^12^ score above the Brazilian population cut-off according to schooling on the MMSE^13,14^ and a score of less than 3.41 on the Informant Questionnaire on Cognitive Decline in the Elderly (IQ-CODE).^15,16^

All subjects who voluntarily consented to participate in the study fulfilled the inclusion criteria (CAPEPesq Ethics Committee. Proc n.701/06).

### Materials and procedures

News items extracted from a radio program of a local AM-frequency station (São Paulo) were recorded over a 2-day period. Twelve were selected considering the presumed interest of the participants in health, education and enjoyable everyday topics.

For the study, 12 news items that fulfilled the following criteria were recorded:

1. News items that were not headlines.
2. News that contained information answering questions such as *when, where, who, what, why, how* and containing numbers that would answer *how much / how many* questions. For example, *how old?*
3. News that did not present technical terms and which was interesting to all participants involved in the study.
4. News which lasted approximately 90 sec.
5. News items which were unrelated to one another.

To select the information to be recorded, four news items were transcribed and divided into propositions corresponding to the following categories: reasons (why/ how); numbers (how much/ how many) and ideas (what, where, who). The most frequent idea was chosen as the key word of the news and three additional more-frequent ideas were considered as the main ideas (main category). The details and the numbers appeared in the news item only once.

Eleven questions were devised for each news item, and each was related to one of the above-mentioned categories. Of the four possible alternatives given, the correct answer directly responded to a particular excerpt of the news while the other three referred to context.

Twelve news items (three targets and nine controls) were presented in three groups of four news items each, with one being the target and other three controls. The main idea could appear in three positions: beginning middle or end, to evaluate the effect of primacy and recency.

There was a five-second interval between the presentation of the news items during which, instructions such as *the second news item follows* were recorded.

The presentation order was not randomized. All subjects heard the news in the same order. The participants were instructed to listen to the four news items, paying equal attention to each, and were informed that upon completion they would be asked questions about one of them. Prior to the application of the questionnaire related to the target news, the examiner presented a sheet of paper containing the key word of the news item in question, and pointed to the key word of the target news saying: “now you will have to answer questions about this news item”.

**Figure 1:**
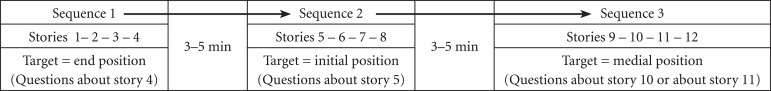
Scheme of stimulus presentation.

In other words, for each sequence of four news items the questions were related to only the target. Three to five minutes later, a new sequence was initiated (Figures 1,2).

**Figure 2:** Example of key words in news item.

University hospitals
Copacabana palace hotel
False registers
Schools

The period between the recording and presentation of the news to the participants was approximately 20 days.

All the data were analyzed statistically using the Bio-Stat application version 3.0. Spearman’s correlation analysis was applied. The level of significance adopted was 5%.

## Results

The mean age of the group was 73.56 yrs (standard deviation=6.26; range=61–83 yrs), and mean years of education was 8.6 yrs (standard deviation=4.41; range=4–19). The mean scores obtained on the MMSE, GDS and IQCODE were 33 (standard deviation=1.64; range=29–35), 5.73 (standard deviation=2.71; range=1–10) and 3 (standard deviation=0; range=2.43–3.37), respectively.

The correct answers in the sequence of four news items summed: sequence 1=10.66 (standard deviation=0.84); sequence 2=10.9 (standard deviation=10.33); sequence 3=10.33 (standard deviation=0.63).

The correlations are presented in Table 1.

**Table 1:** Spearman's correlations between sociodemographic and cognitive variables.

Variable Pairs	Correlation coefficient	Significance (p)
Age × Schooling	-0.408	0.025
Age × MMSE	-0.399	0.029
Schooling × MMSE	+0.454	0.012
Age × Seq.1	+0.156	0.411
Age × Seq.2	+0.084	0.660
Age × Seq.3	-0.043	0.824
Schooling × Seq. 1	+0.165	0.383
Schooling × Seq.2	-0.084	0.658
Schooling × Seq.3	-0.161	0.395

Seq, sequence; MMSE, Mini-Mental State Examination; Level of significance: 0.050.

## Discussion

In relation to age and schooling, the older the individual, the less schooling he/she had, but this interaction of socio-demographic effects did not influence the performance on specific tasks.

Age correlated negatively with MMSE. It is noteworthy that our individuals obtained scores within levels of normality, which does not permit us to interpret any variation as a loss, even though this can occur in the course of cognitive decline related to the normal aging process^22^.

Low scores for schooling were found on the MMSE, where the greater the schooling the higher the score. The relationship between schooling and cognitive decline has been previously described in the literature.^14,17^ Less exposure to formal instruction has been linked to poorer meta-cognitive analysis, comprehension of spoken language and short-term memory, as well as difficulty in visual treatment of information and lower results on tests measuring global intelligence.

The performance in answering the questions was not influenced by subject age or schooling. This is an especially interesting aspect of the study attesting to the functionality of healthy aging in high cognitively demanding situations.

Wingfield^18^ studied these compensating possibilities for comprehension of complex phrases in the elderly population. Recently, another study^19^ identified neurobiological support in the comprehension of narratives, demonstrating the importance of partner familiarity, world knowledge and integration between gestures and language in text comprehension. These clues are available in natural situations, but do not occur in meta-cognitive situations that place additional demand on cognitive resources.

Additionally, high-demand situations include time pressure, competing attention resources and stimuli complexity (non-canonical phrases) for a response. Furthermore, unfavorable contexts include those in which it is not possible to integrate verbal and non-verbal information whether due to unavailability, such as in the case of listening to news on the radio, or due to manipulation of the visual stimuli in the laboratory. Listening without the presence of the speaker represents one such difficult situations.^20^

In face-to-face communication the partner’s hands, corporal and facial clues are available, which increase and integrate the verbal information.

Ryan^11^ highlighted the importance of individual history and experience in performing cognitive tasks. Natural situations are among those more frequently experienced by the aged, which would explain the developing of compensation strategies.

In the situation proposed in the present study, where individuals listen to the reading of sequential news items without the presence of narrator, the four narratives need to be memorized as there is no prior knowledge of the target narrative available, on which the individual will be subsequently questioned.

The individual had no prior knowledge of the news items, which referred to various themes, thus ruling out top-down processing. We believe that no top-down processing occurred, because the news items were not headlines and the time elapsed between the recording and the presentation was greater than 20 days.

Furthermore, the fact that items were read also restricted redundancy and repetition, a facilitating resource for comprehension.^21^ Another factor to be considered is that questions about narratives were presented orally to recognize the correct answer among alternatives in a multiple choice format. Thus, although recognition and not active recuperation of information was requested thereby facilitating the task, it was made more difficult by the high-attention demand and memorization necessary for language processing.

Our aged presented a ceiling effect in their answers. There was a high percentage of correct answers both for main information questions and details, which led us to infer that the aged had appropriately understood and memorized the material.

The heterogeneity of behavior and the possibility of a performance equal or superior to the younger subjects is not a surprise.^22^ Our sample represents a sub-group with high performance in this task.

It should be noted that group median schooling of greater than 8 years may have influenced the good performance of the participants.

In the event of difficulty in working memory, the individual would probably opt to comprehend or memorize the first line of the news item presented. Authors have observed the primacy effect in the healthy aged.^23,24^ However, neither primacy or recency was observed in our study.

The effect of aging on the performance of the MMSE did not manifest in the performance answering questions about the news items. This behavior can be explained by the meta-cognitive characteristic of the MMSE for listening, different than listening to news items that can be included among frequent quotidian tasks especially among aged with median-superior years of schooling.

Another possible explanation is that in natural situations, these aged are capable of compensating any losses that occur in aging. Narrative comprehension is a dynamic and complex activity demanding the participation of both hemispheres. As such, inter and intra-systemic compensations can be mobilized in the comprehension process.^8,25^

The main limitations in our investigation should be noted: the first refers to the fact that we did not have older groups or those with less schooling available to us. Such groups could offer greater detail about possible influences of these variables and would have made our results more representative of the aged population. Other limitations include the small number of participants. Future avenues of this study envisaged include manipulation of the news in order to study attention, working memory and specific language processing, such as information integration in different narrative models.

## Annex

### News item 1

The Secretary of Health guarantees that there is a solution to the crisis of the university hospitals. According to Humberto Costa, the government is presently studying a series of measures to improve the finances of the sector. He reminds that this year, the funds destined to the hospitals increased from 60 to 100 million Reals. Mr. Costa explains, however, that a large part of this money comes from the Secretary of Education.The Health Secretary further explains that there is negotiation currently taking place for a support program with Caixa Econômica Federal Bank. Humberto Costa expects hospitals to make use of these funds for administrative changes. The Dean of the Faculdade Federal de Uberlândia (Federal Faculty of Uberlândia) affirms that the crisis of the university hospitals is the result of a lack of finances. According to Arquimedes Diógenes, for the past eight years the Government has not supplied a reasonable amount of resources for the Health Centers. He further states that the repositioning of professionals who leave the system is accomplished through intermediary action of the so-called Support Foundations. Arquimedes Diógenes underscores that the hospitals must improvise to pay the salaries. The Dean of the Universidade Federal de Uberlândia (Federal University of Uberlandia) expects the Lula government to help the universities pay their debts. Arquimedes Diógenes defends the setting up of an emergency program by Caixa Econômica Federal and BNDES Banks (**time duration: 90 s / radio broadcast: Eldorado AM**).

**Key word:** university hospitals.

**Questions:**

1. How much of an increase did the Government grant to university hospitals? (**Number category**)
  - 50 million reals
  - 60 million reals
  - 100 million reals
  - 150 million reals
2. Where does a substantial part of the money used to increase the funds come from? (**Main information category**)
  - Secretary of Health
  - National Development Bank
  - Caixa Econômica Federal
  - Secretary of Education
3. With which institution has the Secretary of Health been trying to negotiate a support program? (**Details category**)
  - Banco do Brasil
  - Caixa Econômica Federal
  - Federal University of Uberlândia
  - National Development Bank
